# Curating gene sets: challenges and opportunities for integrative analysis

**DOI:** 10.1093/database/baz036

**Published:** 2019-03-19

**Authors:** Jason Bubier, David Hill, Gaurab Mukherjee, Timothy Reynolds, Erich J Baker, Alexander Berger, Jake Emerson, Judith A Blake, Elissa J Chesler

**Affiliations:** 1 The Jackson Laboratory, Bar Harbor, ME, USA; 2 Institute of Biomedical Studies, Baylor University, Waco, TX, USA

## Abstract

Genomic data interpretation often requires analyses that move from a gene-by-gene focus to a focus on sets of genes that are associated with biological phenomena such as molecular processes, phenotypes, diseases, drug interactions or environmental conditions. Unique challenges exist in the curation of gene sets beyond the challenges in curation of individual genes. Here we highlight a literature curation workflow whereby gene sets are curated from peer-reviewed published data into GeneWeaver (GW), a data repository and analysis platform. We describe the system features that allow for a flexible yet precise curation procedure. We illustrate the value of curation by gene sets through analysis of independently curated sets that relate to the integrated stress response, showing that sets curated from independent sources all share significant Jaccard similarity. A suite of reproducible analysis tools is provided in GW as services to carry out interactive functional investigation of user-submitted gene sets within the context of over 150 000 gene sets constructed from publicly available resources and published gene lists. A curation interface supports the ability of users to design and maintain curation workflows of gene sets, including assigning, reviewing and releasing gene sets within a curation project context.

## Introduction

Biocuration plays a central role in biomedical research and public data resources, such as the Gene Ontology (GO). Until recently, curation efforts in functional genomics have focused primarily on gene-by-gene associations. The advent of high-throughput technologies and explosion in biological data have led to the need for collaborative and genome-scale curation.

The GeneWeaver (GW) resource functions as both a multi-species, heterogeneous data store and an analysis platform (1). It is designed to harmonize results from disparate experimental systems and functional genomics data including, but not limited to, differential expression profiling, genome-wide association studies (GWAS), gene networks and literature curation.

Curated, empirical gene sets are supplemented by data automatically aggregated from public resources such as ontological annotations by term from GO, Disease Ontology and Mammalian Phenotype Ontology (MP); pathway databases [e.g. Kyoto Encyclopedia of Genes and Genomes (KEGG) and Pathway Commons (PC)]; and curated repositories (e.g. Comparative Toxicogenomics Database and Molecular Signature Database). Table 1 lists the public resource data that are currently used by GW to create these additional gene sets.

**Table 1:** Public data resources incorporated into GW data store

**Public resource**	**Description**	**Available data sets**	**Reference**
Allen Brain Atlas	Differential expression in adult mouse brain structures	802	(5)
Comparative Toxicogenomics Database	Curated chemical–gene interactions	21 630	(6)
Drug Related Gene Database	Addiction-related experiment data	250	(7)
GO	Gene annotations	85 573	(8)
GWAS Catalog	GWAS summary results	3389	(9)
Human Phenotype Ontology	Gene annotations	6276	(10)
KEGG	Gene network and pathway members	1339	(11)
MeSH	Gene annotations	12 069	(12)
MP	Gene annotations	7931	(13)
Molecular Signatures Database	Curated gene sets	3738	(14)
Online Mendelian Inheritance in Man	Curated disease–gene associations	738	(15)
PC	Gene networks and pathways	1149	(16)

GW recognizes the importance of maintaining current data repositories for reference and data integration. GW supports updates of core species data from model organism databases, NCBI and Ensembl on a quarterly basis. Ontology annotation, UniGene data, probe date reference sets and transcript data are updated bi-monthly or as requested. Furthermore, on a nightly basis, GW creates a data source page, which displays the amount of data contained within the resource and relevant timestamps. This page can be found at https://www.geneweaver.org/data and is linked through the software and in the help documentation. GW currently supports 10 species, but additional species may be added upon request at geneweaver.web@gmail.com.

In addition to its function as a data repository, GW also exposes a suite of analysis tools that users can use to investigate public and curated gene sets. These tools, which operate over collections of gene sets, are based on statistical, combinatoric and graph-theoretic algorithms (1). This allows users to analyze their own experimental data in the larger context of curated, resource-level data sets.

While there are examples of metadatabases or integrative resources that seek to aggregate studies related to disease or genes and provide analysis tools such as MARRVEL (2), ENRICHR (3) and the Open Targets Platform (4), here we report the design and building of a distinctive curation interface into GW. This interface allows groups of curators to manage all aspects of the curation workflow for publications and pre-publication research data including task assignment, literature triage, data import and metadata association. Here we describe the use of GW to curate literature-supported gene sets and give a practical example of how curation of a body of literature can be used to query biological information.

### Capture of gene sets into GW environment

The primary goal of curating literature into GW is to transform published, experimentally supported data into high-quality sets of genes that can be used for computational analysis in the context of gene sets from many sources. Once an article is identified as containing relevant data sets for study, determining how to bin the genes studied in the paper into sets, choosing the most accurate identifiers and documenting relevant metadata become paramount. A general curation strategy is to create separate gene sets for each relevant area of biology in the paper. For example, if the authors report on genes that are up- and down-regulated under two different biological conditions, the creation of four gene sets is appropriate (e.g*.* up-regulated condition 1, up-regulated condition 2, down-regulated condition 1, down-regulated condition 2). Sometimes authors will bin genes in a manuscript according to not only experimental results, but also with respect to some other aspect pertinent to the biology of those genes, such as ‘upregulated transporters’. In those cases, additional gene sets may be created. The general curation guideline for the curation of a gene set is that a set should be created if it relates to a significant piece of biology where it would be useful to compare with other sets. There is no limit to the number of genes appropriate in a set, but creation of intersection and union sets (e.g. all differentially expressed genes, both up- and down-regulated) is not necessary because those can be derived within the GW environment.

**Table 2:** Gene identifier types and expression microarray platforms supported in GW

**Identifier type**	**Supported species**	
CGNC	*Gallus gallus*	
Ensembl gene	All	
Ensembl protein	All	
Ensembl transcript	All	
FlyBase	*Drosophila melanogaster*	
Gene symbol	All	
HGNC	*Homo sapiens*	
MGI	*Mus musculus*	
miRBase	All	
NCBI gene	All	
RGD	*Rattus norvegicus*	
SGD	*Saccharomyces cerevisiae*	
Unigene	All	
Wormbase	*Caenorhabditis elegans*	
ZFIN	*Danio rerio*	
**Expression microarray platforms**		
**Manufacturer**	**Supported species**	**No. of available platforms**
Affymetrix	*Caenorhabditis elegans, Drosophila melanogaster, Danio rerio, Homo sapiens, Macaca mulatta, Mus musculus, Rattus norvegicus, Saccharomyces cerevisiae*	42
Agilent	*Mus musculus, Rattus norvegicus*	4
Illumina	*Homo sapiens, Mus musculus*	4

GW uses a tab-delimited, two-column format for uploading gene sets as bulk lists. The first column contains the gene identifier. We use organism-specific, persistent gene identifiers when possible, generally obtained from the authoritative model organisms database (MOD) resources, due to the ephemeral nature of gene names and symbols. A unique identifier per gene entity is essential because stability of entity recognition is necessary to reference genomic features independent of genome build or exact genomic location or changes in gene nomenclature. To maintain currency with the MOD resources, GW is frequently updated and seamlessly displays the current gene symbols for data. GW also accepts a variety of standard identifiers and aliases for non-gene data sets such as molecular identifiers or microarray probe identifiers (Table 2). For empirical data, it is preferable to choose identifiers that reflect precisely what was measured in the experiment (e.g. hybridization to a particular probe sequence), rather than what gene was thought to be targeted by that probe at the time of publication or accession. The current best gene mappings for these probes are stored in the GW system and used in analyses.

**Figure 1:**
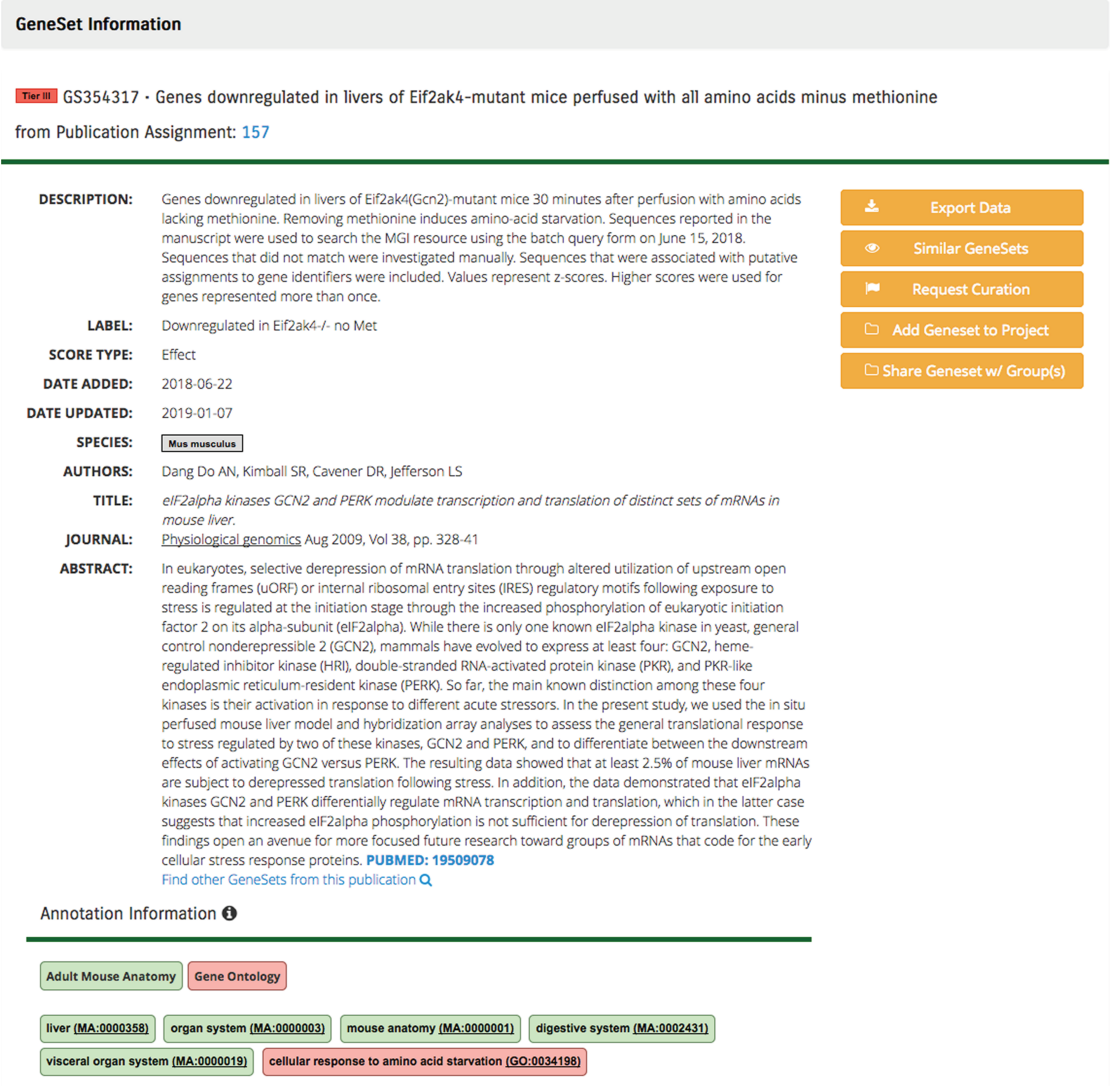
Sample GeneSet from GW showing the curator-populated fields of Gene Set Name, Gene Set Figure Label. Gene Set Description and Ontology Annotations. Publication data are populated by providing the PMID during the set upload.

The second column in a GW ‘GeneSet upload’ is for a data value or score type. Priority is given to the statistics that are used to associate a particular gene to the GeneSet. This field can also be used to represent values associated with a quantitative measurement of the data such a fold change or z-score. If complete genomic data are available, they are uploaded although the user has the option of filtering significant data in an analysis. The priority for the type of data to include in column two is *Q*-values, *P*-values, effect size, followed by fold change and finally binary (indicating presence or absence in a GeneSet).

A practice central to gene set curation is adherence to metadata standards. There are certain situations that arise that, based on GW curation guidelines, a curator uses their scientific judgment to accurately capture the appropriate data from a publication. When, for example, more than one result is reported for a gene, a common occurrence with legacy microarray data, a consistent approach must be taken in capturing that data. In this situation, the curator would report the gene once, populating column two with either the strongest statistical association score or with the highest fold change value associated with the identical gene(s). In other situations, the fold change for a given gene is not reported as a value but as infinite fold change usually because in one of the reference samples the gene was undetectable. In these cases, the curator chooses a specific value for the gene–value pair to distinguish this situation. Any of these kinds of rules used in the curation of a gene set will be noted in the gene set description. These rules are described in the curation standard document (http://geneweaver.org/help/) and are mentioned in the GeneSet descriptions.

Creation of a gene set also requires capture of additional metadata that is used for searching and the classification of the gene set within the GW environment (Figure 1). The following fields are necessarily included with the curation of a gene set from a curated manuscript:

**Figure 2:**
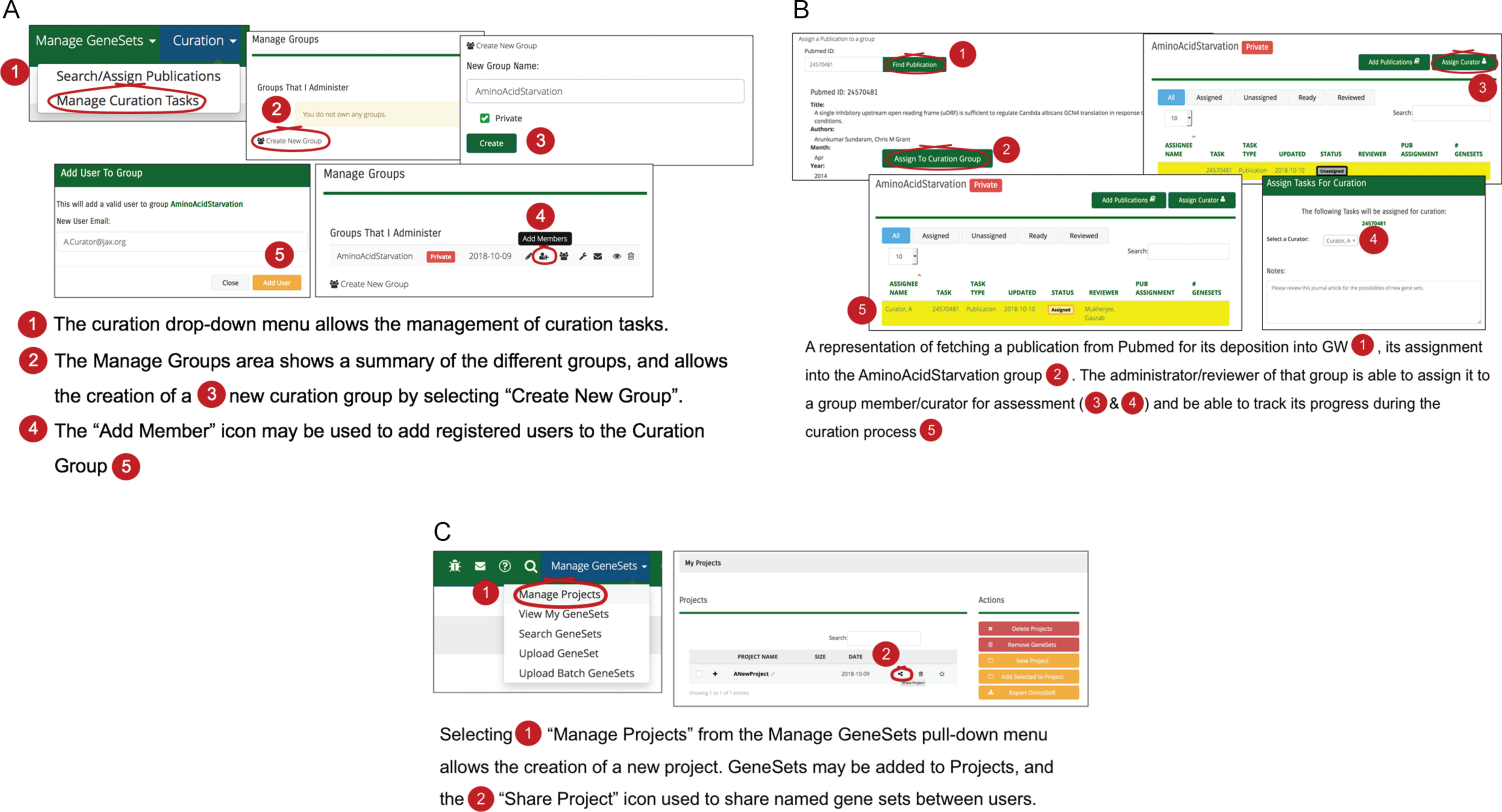
**(A)** A workflow diagram representing the creation of a new curation group. **(B)** A diagram showing the management of curation tasks and their assignment to group members. **(C)** The creation of a new project and the subsequent assignment of gene sets to it, then allows the sharing of that gene set among users.

**Gene Set Name:** a brief title for the gene set, approximately sentence length, which should provide a clear and concise description of the contents of a gene set interpretable to most users of GW, but with sufficient detail to satisfy a domain expert. This is the major gene set name that is displayed in all search results, project directory and table views of analysis results.

**Gene Set Figure Label:** a brief 23-character abbreviation to facilitate recognition of the gene set in a graph, analysis result or other display.

**Gene Set Description**: a detailed description of the gene set, including rules for its construction, experimental methods and analyses used to generate data, anatomical terms and traceable references to source data including accession information and date. Abbreviations should be avoided.

**Ontology Annotations:** ontology annotations are assigned to gene sets. These appear under the heading of annotation information. Terms from Disease Ontology, Mammalian Phenotype Ontology, Adult Mouse Anatomy, EMBRACE Data and Methods, Chemical Entities of Biological Interest, Medical Subject Headings (MeSH), Experimental Factor Ontology and Human Phenotype Ontology are provided. This is facilitated through the application of the Monarch or NCBO Annotator to provide textual descriptions, including publication abstracts and manually reviewed. Curators may assign terms to gene sets outside of the recommended terms and remove false-positive recommendations.

**Publication Information:** PubMed ID, Title, authors, publication information and full text of the abstract.

### Curation workflows and strategies

The curation pull-down menu in GW provides various strategies for managing curation tasks such as searching and assigning publications from peer-reviewed journals. The workflow process allows the creation of a curation group, i.e. a group of curators that have GW editorial accounts (Figure 2A). A lead curator can assign curation tasks to members of the curation group, typically assigning publications for review and gene set entry into the resource to specific curators. Once the gene sets are created for a curation task, the lead curator may choose to review each gene set prior to approval (Figure 2B). Once accepted, the gene set becomes available based on its sharing restrictions. Sharing restrictions can be set that allow data sets still under preliminary analysis by a research group to be private, all the way to having data sets that are freely and publicly available. Once public, a gene set may be added to a ‘project’ and used for analysis (Figure 2C). A project typically consists of a set of related gene sets that have been defined within the GW environment concerning a biological hypothesis that is under investigation. Related gene sets may be grouped into projects that can be shared publicly or within analysis teams.

**Figure 3:**
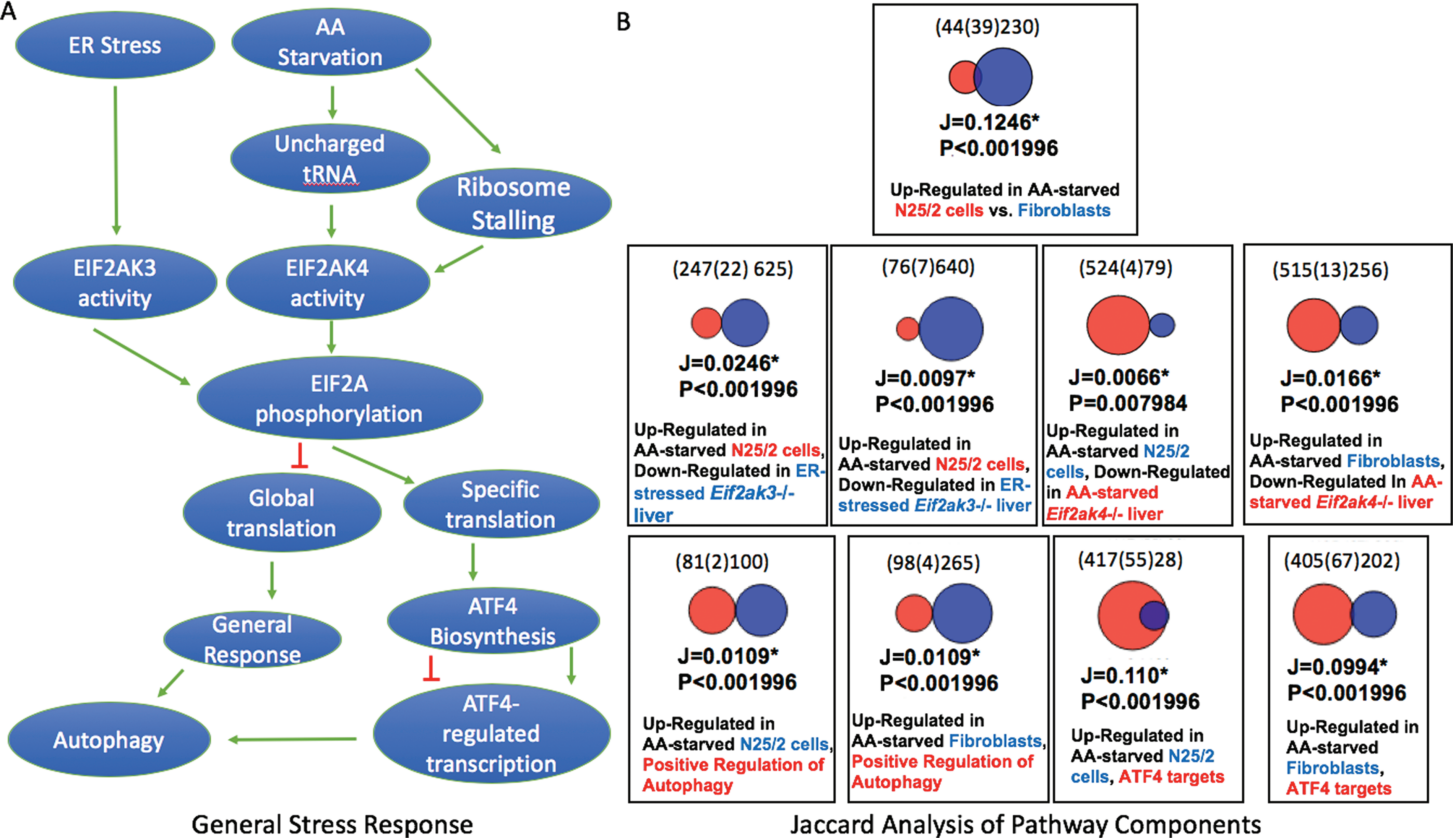
(**A**) A schematic representation of two branches of the ISR. Unfolded protein ER stress or amino acid starvation activates two separate kinases, EIF2AK3 or EIF2AK4, which phosphorylates EIF2A. Phosphorylated EIF2A then represses general translation in the cell, but stimulates translation of a subset of response genes including the transcription factor ATF4. A consequence of ATF4 activation is the downstream activation of autophagy. (**B**) The results of Jaccard similarity analyses using independently curated gene sets that represent different aspects of the ISR. These results show significant similarity between genes up-regulated after amino acid starvation and genes down-regulated in EIF2A kinase mutant cells, ATF4 target genes and genes annotated with the GO term ‘positive regulation of autophagy’ (GO:0010508, downloaded from a Mouse Genome Informatics Query performed on 17 August 2018).

**Table 3:** Gene sets used for analysis of the ISR

**Gene set identifier**	**# of genes**	**Gene set title**	**Reference**
GS355023	102	Positive regulation of autophagy	(8)
GS354317	528	Genes down-regulated in livers of Eif2ak4-mutant mice perfused with all amino acids minus methionine	(23)
GS354521	647	Genes down-regulated in livers of Eif2ak3-mutant mice treated with tBuHQ	(23)
GS354534	83	Genes up-regulated in amino acid-starved N25/2 cells	(24)
GS355584	472	Atf4 target genes	(25)
GS354663	269	Genes up-regulated in amino acid-starved fibroblasts	(26)

### Curated sets can be used to investigate relationships between biological processes

To assess the utility of expert curation into the GW resource, we focused on a well-known pathway i.e. the integrated stress response (ISR) pathway, curating genes sets relevant to the execution of the pathway and comparing those gene sets using the Jaccard analysis tool. Jaccard statistics are widely used to estimate the similarity of sets and originate from studies of species habitation of ecological zones. Briefly, these statistics estimate the positive match of categorical data and are considered a preferred alternative to set matching methods that are biased by the large number of negative results that are often found in comparison of genomic data sets. The simplest Jaccard statistic takes the intersection size over the size of the union of two gene sets. In GW, the size of the union is based on the possible matches between two gene sets, such that gene products that could only be present in one set are excluded, e.g. genes for which there is no known orthologue.

Our hypothesis was that curation of a group of gene sets that represent gene expression associated with steps of a well-defined pathway would result in significant Jaccard overlap of the gene sets. The ISR pathway is a well-conserved and well-studied pathway that is amenable to this type of analysis (17). Two branches of the ISR are shown in Figure 3A. The pathway begins with the detection of a cellular stress such as amino acid starvation or unfolded proteins that then activate a protein kinase that phosphorylates EIF2A (18, 19). The phosphorylation of EIF2A results in the repression of global translation, but the activation of a small set of genes that coordinate the response (20). One of those genes is ATF4, which regulates transcription of downstream response genes (21). Some of those genes in turn up-regulate autophagy (22). To illustrate the value of curated gene sets in the GW resource, we independently curated sets that were relevant to the ISR. The sets included genes that were up-regulated after amino acid starvation in two cell lines, genes that were down-regulated in amino acid-starved *Eif2ak4* (GCN2) mutant livers, genes that were down-regulated in endoplasmic reticulum (ER) stress *Eif2ak3* (PERK) livers, genes that bound the ATF4 transcription factor within 3 kb of their promoters and mouse genes that are annotated to the GO term ‘positive regulation of autophagy’ (Table 3). Our results show that the gene sets up-regulated in the amino acid-starved cells overlapped significantly with each other, *P* < 0.002 (the upper limit value reported by GW) and overlapped significantly with the gene sets identifying the various downstream steps of the ISR, *P* < 0.002.

## Conclusions

GeneWeaver in contrast to many bioinformatics resources, provides a curation component that focuses on gene sets as the reportable entity. Working from both the data-download and the literature-curation aspects, GW now comprises over 150 000 high-quality gene sets that are accessible for data analysis. Selected gene sets can be defined and analyzed within the GW analysis platform, and/or provisional gene sets can be analyzed in a restricted access environment. The ability to compute on gene sets from multiple resources is essential in today’s genome systems and comparative functional analysis environments. These analyses allow experimental data to be immediately placed in the context of other experiments and of rigorously curated pathways and gene functional annotations.

## Notes

**Database URL:** https://www.geneweaver.org/
